# Comparative Efficacy of Intraoperative Patient State Index vs. Bi-Spectral Index in Patients Undergoing Elective Spine Surgery with Neuromonitoring Under General Anaesthesia: A Randomized Controlled Trial

**DOI:** 10.4274/TJAR.2024.241663

**Published:** 2024-09-17

**Authors:** Deepak Singla, Sanjay Agrawal, Priya TK, Anirban Brahma Adhikary, Mishu Mangla

**Affiliations:** 1 All India Institute of Medical Sciences, Department of Anaesthesiology, Rishikesh, India; 2 All India Institute of Medical Sciences, Department of Anaesthesiology and Critical Care, Jodhpur, India; 3 All India Institute of Medical Sciences, Department of Obstetrics and Gynaecology, Bibinagar, Hyderabad, India

**Keywords:** Consciousness monitors, electroencephalogram, intravenous anaesthesia, intraoperative monitoring, neuroanaesthesia, spine

## Abstract

**Objective:**

Various electroencephalogram-based monitors have been introduced to objectively quantify anaesthesia depth. However, limited data are available on their comparative clinical efficacy in various surgical procedures. Therefore, we planned this study to compare the relative efficacy of patient state index (PSI) vs. Bi-spectral index (BIS) assessment in patients undergoing elective spine surgery under general anaesthesia.

**Methods:**

This prospective, parallel-group, single-center study included patients undergoing major spine surgery with neuromonitoring. Patients were randomized into two groups, i.e., group B (undergoing surgery under BIS monitoring) and group P (undergoing surgery under PSI monitoring). The primary objective was to compare the time to eye opening after stopping anaesthetic drug infusions.

**Results:**

The mean propofol dose required for induction in group B was 130.45±26.579, whereas that in group P, it was 139.28±17.86 (*P* value 0.085). The maintenance doses of propofol and fentanyl required for surgery were also comparable between the groups. Time to eye opening was 12.2±4.973 in group B and 12.93±4.19 in group P, with a *P* value of 0.2664 (U-statistic-684.50).

**Conclusion:**

The intraoperative PSI and BIS had similar clinical efficacy in terms of the dose of propofol required for induction, time of induction, maintenance dose of propofol and fentanyl, time of eye opening, and recovery profile in patients undergoing elective spine surgery under neuromonitoring.

**Main Points**

• Both Bi-spectral index (BIS) and patient state index (PSI) have been used and validated for monitoring the depth of anaesthesia but have not been extensively studied in patients requiring intraoperative neuromonitoring.

• Our study found that using either BIS or PSI resulted in similar clinical efficacy in terms of intraoperative anaesthetic consumption and postoperative recovery in patients requiring intraoperative neuromonitoring.

• Therefore, either of the two approaches can be used to monitor the depth of anaesthesia during the intraoperative period in this group of patients.

## Introduction

The assessment and maintenance of adequate anaesthesia depth has remained an important clinical consideration for anaesthesiologists.^1^ Inadequate depth of anaesthesia can lead to intraoperative awareness, whereas excessive use of anaesthetics can cause delayed awakening and wastage of anaesthetic drugs. Clinical parameters, like heart rate, blood pressure, and lacrimation, provide subjective assessments but are grossly inadequate for quantifying the depth of anaesthesia. Recently, various electroencephalogram (EEG) based monitors have come into use to objectively quantify the depth of anaesthesia.^2^ Most of these monitors use the frequency and wavelength of specific EEG waveforms and provide an assessment of depth in the form of a number ranging from 0 to 100.^2^

The most commonly used EEG-based anaesthesia depth assessment monitor is the Bi-spectral index (BIS). BIS was the first monitor approved by the Food and Drug Administration for this purpose.^3^ It uses a single-channel frontal EEG and assesses awareness considering various parameters like frequency, phase, and power spectrum, as a dimensionless number. The normal range for BIS for adequate depth of anaesthesia is 40-60. Its mathematical algorithm responds rapidly to changes in EEG frequency and thus provides rapid evaluation of anaesthesia depth.^4^

The patient state index (PSI) is a more recent depth of anaesthesia monitor that relies on 4-channel EEG following advanced artifact removal.^5^ The normal range of PSI for adequate depth of anaesthesia is 25-50. Compared with conventional EEG monitoring, PSI signals are shown to be less affected by electromyography (EMG) and thus may be better in patients in whom EMG may interfere with an adequate assessment of depth of anaesthesia. Studies have also shown that compared with PSI, BIS reacts faster to changes in sevoflurane concentration.^6^ However, after a thorough search of the literature, we were not able to find any studies comparing BIS with PSI in patients undergoing spine surgery under total intravenous anaesthesia, in terms of the anaesthetic dose used for induction and maintenance, recovery profile, and incidence of complications like awareness and recall under anaesthesia. Therefore, this study was planned by us to compare the relative efficacy of PSI vs BIS assessment in patients undergoing elective spine surgery under general anaesthesia.

## Methods

This was a prospective, parallel-group, single-center randomized control study approved by the Institutional Ethical Committee of All India Institute of Medical Sciences, Rishikesh (Uttarakhand) (decision no.: AIIMS/IEC/19/749, date: 12.04.2019) and registered in the clinical trial registry (CRTI) of India (CTRI/2021/12/038503). We followed the Helsinki Declaration of 1964, revised in 2013. The inclusion criteria for this study were age 18-70 years and American Society of Anesthesiologists class I or II who underwent major spine surgery with neuromonitoring under general anaesthesia. The exclusion criteria included patients with clinically significant cardiovascular, respiratory, hepatic, renal, or metabolic disease, alcohol or drug abuse, use of regional/neuraxial blocks for intraoperative analgesia, neurological or psychiatric disorders, and use of antiepileptic or other centrally acting drugs.

Informed written consent was obtained from all patients before enrollment in this study. A computer-generated randomization sequence was used to randomize patients into groups B and P based on simple randomization and 1:1 allocation. Group B patients underwent spine surgery under BIS monitoring [BIS LOC2 channel (Coviden)], maintaining a BIS value between 40 and 60 during the intraoperative period, and group P patients underwent spine surgery under PSI monitoring [SEDLine on Root monitor (Masimo)], using a range of 25-50 for anaesthesia. Baseline awake mean arterial pressure (MAP), heart rate, and BIS/PSI were noted in both groups. After that, patients were anesthetized with propofol at an infusion of 30 mg kg^-1 ^hr^-1^ i.v. until there was loss of response to the eyelash reflex and desired BIS/PSI values were achieved in the respective groups. A maintenance infusion of propofol was started at 2 mg kg^-1 ^hr^-1^ and titrated to maintain a BIS value of 40-60 in group B and a PSI value of 25-50 in group P. Subsequently, fentanyl 2 µg kg^-1^, vecuronium 0.1 mg kg^-1^were given, and intubation was performed after 3 min of positive pressure ventilation in both groups.

No further vecuronium dose was administered. Surgery was performed under continuous infusion of propofol and fentanyl (started at 1 µg kg^-1 ^hr^-1^) titrated to maintain BIS/PSI values in the above-mentioned range in both groups. MAP, heart rate, MAC, and BIS/PSI were recorded after induction, intubation, and surgical incision on an hourly basis until completion of surgery. Postoperatively, patients were administered neostigmine and glycopyrrolate for relaxation reversal, and BIS/PSI values were noted at the time of eye opening and extubation. The modified Observer’s Assessment of Alertness and Sedation (mOAAS) was noted before and after extubation. Time to eye opening after switching off the propofol infusion was noted in both groups. Patients were also assessed for intraoperative awareness or recall immediately and 24 hours after surgery.

The primary objective of this study was to compare the time to eye opening after stopping anaesthetic drug infusions in both groups. The secondary objectives were to compare the dose of propofol required for induction, time of induction, total maintenance dose of propofol and fentanyl used intraoperatively, mean heart rate and MAP in both groups, mOAAS score at emergence, and relative incidence of complications (mentioned below) in both groups.

We noted complications like intraoperative awareness and recall, bradycardia, tachycardia, hypertension, and hypotension. Any intraoperative event occurrence confirmed by OT staff was defined as intraoperative awareness and recall. A heart rate of less than 50 min^-1^ was defined as bradycardia, whereas a heart rate of more than 100 min^-1^ was defined as tachycardia. Hypotension was defined as a fall in the MAP by more than 25% from the baseline value or any value of less than 55 mmHg. Similarly, hypertension was defined as MAP >100 mmHg or a rise >25% from the baseline value. In cases of bradycardia, in. atropine 0.6 mg of inj. atropine was used. For hypotension inj,. mephentermine 6 mg i.v. was used. For hypertension and tachycardia, an fentanyl bolus of 0.5 µg kg^-1^ i.v. was used.

### Study Sample Size

Since there have been no similar studies in the past comparing the time of eye opening after stopping anaesthetic drug infusions with BIS and PSI, we used an effect size of 0.6 (medium effect size using Cohen’s convention). The sample size was calculated using G*Power software version 3.1.9.7, comparing two different means using a one-tailed analysis. To achieve 80% power of the study with an alpha error of 0.05, we need to have 36 participants per group. Therefore, we included 40 patients in each group (a total of 80 patients) to compensate for any dropouts.

### Statistical Analysis

The data was analyzed using Statistical Package for the Social Sciences (SPSS v23, IBM Corp.). Mean ± standard deviation was used for continuous data, and number/percentage was used for categorical data. To compare two groups, we used the unpaired t-test, if the data were normally distributed or Mann-Whitney test, if the data were skewed. A *P* value of < 0.05 was considered statistically significant.

## Results

**Figure 1:**
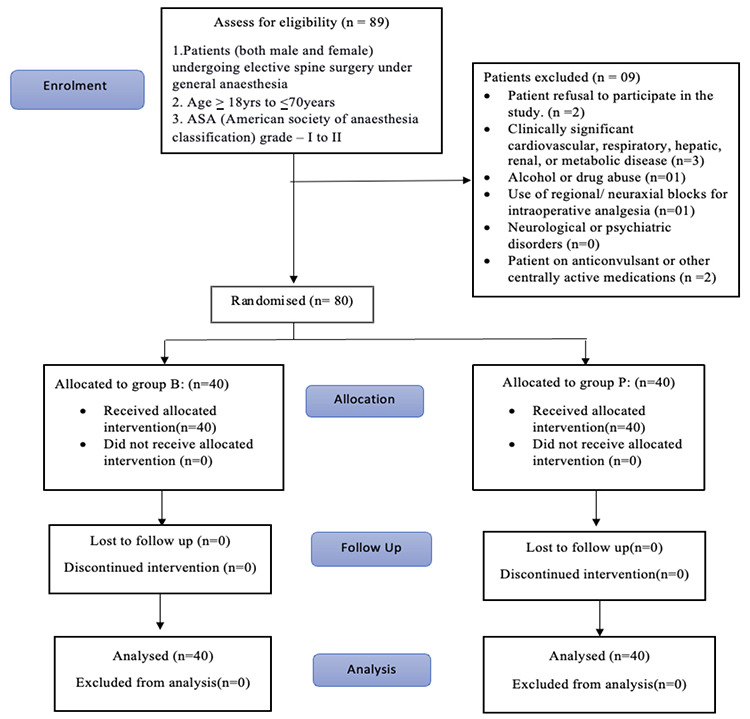
CONSORT Flow Diagram

We included 89 patients in our study. Of these, two patients refused to participate in the study, three had significant comorbidities, one was a known alcoholic, one received erector spinae plane block, and two were on anticonvulsants. Therefore, we enrolled 80 patients in our study (Figure 1). The demographic characteristics of the patients in both groups were comparable (Table 1). The mean durations of anaesthesia and surgery were also similar between the groups (Table 1).

**Figure 2:**
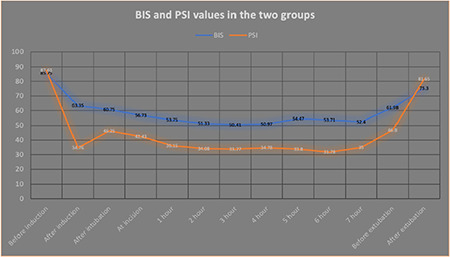
Mean intraoperative BIS vs PSI values in the two respective groups BIS, Bi-spectral index; PSI, patient state index.

The mean dose of propofol (in mg) required for induction in group B was 130.45±26.579, while in group P, it was 139.28±17.86 (*P *value 0.085). The time (minutes) required for induction was 4.025±0.9046 in group B and 4.23±0.3824 in group P, with no statistically significant difference (*P *value 0.1907). The mean heart rate (Table 2) and MAP (Table 3) were analyzed intraoperatively at different time points, and no significant difference was noted in the two groups at any time point. The mean PSI and BIS values were within the acceptable range for both groups (Figure 2) at all time points.

The maintenance doses of propofol and fentanyl required for surgery were also comparable between the groups (Table 4). Time to eye opening was 12.2±4.973 in group B and 12.93±4.19 in group P, with a *P *value of 0.2664 (U-statistic-684.50). The mOAAS scores were also comparable in both groups. The mean mOAAS score before extubation in group B was 1.95±0.8458, whereas that in group P, it was 1.6±0.7442, and the difference was not statistically significant (*P *value 0.0530). After extubation, the mean mOAAS score was 3.225±0.6197 in group B and 3.3±0.6485 in group P, and the difference was again found to be statistically non-significant (*P *value 0.5984).

The incidences of various complications in both groups were comparable. In group B, four patients had bradycardia, three had tachycardia, five had an episode of hypotension, and two had an episode of hypertension. In group P, five patients had bradycardia, three had tachycardia, four had hypotension, and three reported hypertension. No patient exhibited no intraoperative awareness or recall during the surgery.

## Discussion

We conducted a randomized controlled trial of patients undergoing major spine surgery with neuromonitoring under general anaesthesia. Patients were comparable in terms of demographic profile and duration of surgery and anaesthesia. The dose of propofol required for induction was lower in group B than in group P, but the difference was not statistically significant. Similarly, there was a statistically non-significant difference in the time required for anaesthesia induction, which was less in group B. We did not find any statistically significant difference in mean heart rate and MAP between the groups during the intraoperative period. Similarly, the maintenance doses of propofol and fentanyl were similar between the groups. After surgery, the time to eye opening was similar in both groups. The mean mOAAS score was higher in group B at the time before extubation, but the difference was not statistically significant. After extubation, the mOAAS score was comparable in both groups. The relative incidence of various complications was also comparable in both groups.

Two of the most commonly used EEG-based monitoring systems are the BIS and the PSI. While BIS monitors EEG in one hemisphere only, PSI measures EEG in both hemispheres.^7^ A previous study concluded that using PSI to titrate propofol administration resulted in a significantly reduced usage of propofol and improved early recovery profile.^8^ However, in our study, the mean dosage of propofol required for induction was higher with the PSI monitor (139.28±17.86) compared to BIS (130.45±26.579), but the difference was statistically insignificant. In another study, the use of BIS, compared with PSI, showed a decrease in the dosage of propofol required for induction; however, in our study, it was found to be statistically insignificant (*P*=0.085).^9^

The Modified Observer’s Assessment of Alertness and Sedation (mOASS score) is a validated 6-point scale that assesses the responsiveness of individuals and correlates with the ASA continuum of sedation. A previous study compared the correlation of BIS and PSI with mOASS and showed that BIS had a stronger association.^10^ However, in our study, the mOASS score was comparable between the monitors at various time points. No significant hemodynamic changes were noted while using both monitors, which is consistent with a previous study.^11^ A previous study concluded that BIS and PSI are comparable to each other in terms of differentiating consciousness and unconsciousness, during the induction of anaesthesia and emergence, and during any episode of awareness, in patients undergoing surgery, which is consistent with our study.^12^

The mean dose of fentanyl was found to be comparable between the groups in our study. The depth of anaesthesia is also influenced by the level of analgesia. The nociception level index using tetanic stimulation was previously used to titrate remifentanil, but its utility in a surgical setting has not been established.^13^ Therefore, most anaesthesiologists use clinical parameters to titrate opioids.

In our study, there was no significant difference between the two groups in the total intraoperative consumption of propofol. In a study, authors reported that processed EEG values may help clinically evaluate the depth of anaesthesia but do not correlate well with clinical parameters during the period of awakening or deep anaesthesia planes.^14^ Thus, a clinician’s decision to alter an anaesthetic agent may not always correlate well with real-time brain function monitoring. Moreover, processed EEG in many instances may take more time to reflect changes than cardiovascular indices. Further studies are required to establish the time delay between the change in conscious level and the change in processed EEG signals. From our study, we can conclude that both BIS and PSI are equally effective in titrating anaesthetic agents and provide an extra edge over the traditional way of using only clinical parameters like heart rate, blood pressure, and movement to noxious stimuli in response to surgical stimuli as a guide to anaesthesia depth monitoring.

The time of recovery from anaesthesia (time to eye-opening) was similar in both groups in our study. Similar findings were reported by a study,^7^ which found that BIS and PSI values after neuromuscular block reversal were similar in patients undergoing surgery under total intravenous anaesthesia. EEG-based monitors have been successfully used in select patient groups to avoid delayed recovery.^15^ A recent meta-analysis has shown that the use of BIS-guided anaesthesia results in reduced recovery times, reduced anaesthesia agent dose, and reduced risk of adverse events.^16^ According to another meta-analysis, the use of BIS in elderly patients resulted in an improved recovery profile but did not reduce the incidence of postoperative delirium.^17^

In our study, no patient had intraoperative awareness in any group when the BIS and PSI values were in the recommended range. A randomized control trial^18^ similarly found that the use of BIS resulted in decreased awareness in at-risk patients undergoing surgery under general anaesthesia with muscle relaxants. In an extensive systematic review,^19^ the authors concluded that the use of the BIS might reduce the incidence of awareness, but because of the low incidence of incidence, the evidence of the effectiveness of the BIS is not precise.

### Study Limitations

Our study has limitations because it was performed in a specific population of patients who were undergoing spine surgery under total intravenous anaesthesia. Therefore, these findings may not be applicable to patients undergoing other types of surgery under different anaesthetic techniques. Second, the effect site concentrations of propofol and fentanyl were not estimated in our study because the monitors for the same concentrations were not available in our institute at the time of conducting this study. Third, our study did not evaluate the effect of coadministration of other agents like dexmetatomidine. Thus, further studies are needed to determine whether BIS and PSI perform comparatively with different anaesthetic agents and different types of surgeries.

## Conclusion

In conclusion, the intraoperative PSI and BIS had similar clinical efficacy in terms of the dose of propofol required for induction, time of induction, maintenance dose of propofol and fentanyl, time of eye opening, and recovery profile in patients undergoing elective spine surgery under neuromonitoring. Thus, both BIS and PSI can be used in patients with similar outcomes.

## Ethics

**Ethics Committee Approval:** The study approved by the Institutional Ethical Committee of All India Institute of Medical Sciences, Rishikesh (Uttarakhand) (decision no.: AIIMS/IEC/19/749, date: 12.04.2019) and registered in the clinical trial registry (CRTI) of India (CTRI/2021/12/038503).

**Informed Consent:** Written consent was obtained from all patients prior to their inclusion in the study.

## Figures and Tables

**Table 1. Demographic Profile of the Patients in Group B and Group P:** 

**Parameter**	**Group B**	**Group P**	***P* value**
Age; years	44.2*±*17.96	48.525*±*15.39	0.256^‡^
Sex; M:F	25:15	24:16	--
BMI^*^; kg m^-2^	26.44*±*4.43	25.74*±*4.85	0.502^‡^
ASA^†^ grade; I:II	25:15	31:9	--
Duration of surgery; hours	4.47*±*1.29	4.49*±*1.13	0.78^§ ^(U-statistic-768.00)
Duration of anaesthesia; hours	5.04*±*1.33	4.97*±*1.18	0.94^§^ (U-statistic-791.50)

Values are mean ± SD or number (proportion)

^*^BMI, body mass index; ^†^ASA, American Society of Anesthesiologists; ^‡^t-test; ^§^Mann-Whitney U test; M:F, male:female.

**Table 2. Mean Heart Rates in Groups B and P During the Intraoperative Period:** 

**Parameter**	**Group B**	**Group P**	***P* value**
	**Mean ± SD**	**Mean ± SD**	
Before induction	73.92*±*6.61	75.1*±*5.4	0.294^* ^(U-statistic-691.50)
After induction	73.05*±*5.26	73.95*±*6.45	0.234^* ^(U-statistic-676.50)
After intubation	81.65*±*7.33	79.45*±*6.86	0.339^* ^(U-statistic-700.50)
At incision	79.23*±*6.29	76.53*±*5.99	0.0633^* ^(U-statistic-607.50)
1 hour	72.63*±*6.25	73.95*±*6.45	0.1485^* ^(U-statistic-650.00)
2 hours	72.63*±*5.39	73.85*±*5.33	0.346^* ^(U-statistic-702.50)
3 hours	72.56*±*6.44	73.31*±*6.78	0.621^†^
4 hours	72.77*±*6.24	72.18*±*6.58	0.719^†^
5 hours	72.82*±*6.34	70.81*±*8.52	0.446^†^
6 hours	84.57*±*7.28	82.56*±*12.75	0.715^†^
7 hours	79.4*±*7.4	81*±*13.09	>0.999^* ^(U-statistic-9.50)
Before extubation	84.15*±*8.98	87.3*±*8.43	0.097^* ^(U-statistic-628.50)
After extubation	77.83*±*5.03	79.4*±*5.28	0.169^*^ (U-statistic-659.00)

Values are mean *±* SD

^*^Mann-Whitney U test; ^†^t-test; SD, standard deviation.

**Table 3. Mean Arterial Pressure in Groups B and Group P During Intraoperative Period:** 

**Parameter**	**Group B**	**Group P**	***P* value**
	**Mean ± SD**	**Mean ± SD**	
Before induction	72.25*±*5.001	75.425*±*9.109	0.26^* ^(U-statistic-683.50)
After induction	73.85*±*5.691	76.375*±*7.458	0.143^* ^(U-statistic-648.50)
After intubation	80.475*±*11.431	79.95*±*10.891	0.98^* ^(U-statistic-797.00)
At incision	79.075*±*10.341	77.925*±*8.748	0.729^* ^(U-statistic-764.00)
1 hour	77.225*±*6.945	75.975*±*7.458	0.413^* ^(U-statistic-715.50)
2 hours	75.65*±*6.822	73.825*±*6.621	0.316^* ^(U-statistic-696.00)
3 hours	74.103*±*5.651	74.846*±*7.618	0.943^* ^(U-statistic-753.00)
4 hours	75.2*±*7.993	73.812*±*7.785	0.582^* ^(U-statistic-440.50)
5 hours	76.35*±*7.729	75.47*±*9.716	0.779^†^
6 hours	76.86*±*7. 358	71.56*±*12.381	0.306^†^
7 hours	75.6*±*8.325	72.5*±*10.786	0.711^* ^(U-statistic-8.00)
Before extubation	71.725*±*4.552	71.475*±*7.2	0.617^* ^(U-statistic-748.50)
After extubation	75.05*±*7.524	74.025*±*6.083	0.594^* ^(U-statistic-744.50)

Values are mean *±*SD

^*^Mann-Whitney U test; ^†^t-test

**Table 4. Mean Intraoperative Propofol and Fentanyl Consumption in Groups B and P:** 

**Parameter**	**Group B**	**Group P**	***P* value**
Propofol; 1%	19.35*±*7.62	21.38*±*7.89	0.2423^* ^(U-statistic-678.00)
Fentanyl; 10 µg kg^-1^	10.69*±*2.57	9.22*±*1.64	0.0599^*^ (U-statistic-604.00)

Values are mean *±* SD and rate of infusion is in mL hr^-1^

^*^Mann-Whitney U test
